# Normal levels of protein C and protein S tested in the acute phase of a venous thromboembolic event are not falsely elevated

**DOI:** 10.1186/1477-9560-8-10

**Published:** 2010-05-18

**Authors:** Leonard Minuk, Alejandro Lazo-Langner, Judy Kovacs, Melinda Robbins, Bev Morrow, Michael Kovacs

**Affiliations:** 1 Department of Medicine, Division of Hematology, London Health Sciences Centre, London, Ontario, Canada; 2 Schulich School of Medicine and Dentistry, University of Western Ontario, London, Ontario, Canada

## Abstract

**Background:**

Protein C (PC) and protein S (PS) determination is part of the thrombophilia investigation in patients with idiopathic venous thromboembolism (VTE). Based on scarce evidence it is a common notion that PC and PS levels decrease during the acute phase of VTE, necessitating delay of testing and temporary transition from warfarin to low molecular weight heparin. We have previously demonstrated that an abnormal PC or PS result determined within 24 hours of VTE diagnosis and before the initiation of warfarin needs to be repeated for confirmation ≥3 months after starting treatment and ≥14 days after stopping anticoagulation therapy. In the current study, we sought to show that normal PC and PS values determined during the acute phase of VTE are not false negatives.

**Methods:**

99 patients with acute idiopathic VTE who had normal PC and PS determination within the first 24 hours of presentation and who subsequently had their oral anticoagulation discontinued after six months of therapy. PC and PS determinations were repeated ≥6 months after starting treatment and ≥ 14 days after stopping warfarin. Proportions of patients who tested abnormal on the second test were calculated and 95% confidence intervals obtained using the Wilson's score method. Data from a previously published study on patients with abnormal initial tests was included for comparison.

**Results:**

None of the 99 patients who had normal PC and PS initially had an abnormal result on repeated testing (0%; 95% CI 0 - 3.7%). Data from the previous study showed that, among patients who initially had abnormal results, 40% (95%CI 35.4-84.8%) were confirmed to have low PC and 63.6% (95%CI 16.8-68.7%) low PS on repeated testing. The difference between proportions was statistically significant (χ^2 ^p-value < 0.001).

**Conclusion:**

Our results suggest that PC and PS can be determined during the acute phase of VTE and whereas abnormal results need to be confirmed with repeat testing at a later date, a normal result effectively rules out deficiency with only one test.

## Introduction

Venous thromboembolism (VTE) is a common event, often precipitated by surgery, immobility or active malignancy[1]. Many cases, however, have no clear precipitant and are defined as idiopathic VTE [2-4]. The diagnostic work up for these patients includes testing for inherited and acquired hypercoagulable conditions, usually including functional quantitative assays for proteins C and S, and antithrombin, as well as testing for lupus anticoagulant, antiphospholipid antibodies, activated protein C resistance (with or without genetic testing for Factor V Leiden) and determination of the G20210A Prothrombin gene mutation[4]. Although from a practical standpoint this group of tests is most conveniently performed at the time of acute VTE diagnosis, concerns have been raised in the literature by studies suggesting that acute VTE may alter the levels of coagulation factors and lead to false positive (i.e. low) results.

Specifically, it is commonly believed that proteins C and S levels are markedly decreased during the initial phases of VTE, presumably secondary to consumption of these factors, thus rendering them uninterpretable. The evidence that protein C and S levels are decreased during an acute VTE event is based on a study by D'Angelo et al [5]. This was a small series of 8 patients and only reported a lower mean protein C and S level and not the proportion of patients who had an abnormal result. Historically, some consider that protein C and S can also be falsely elevated on the basis of being acute phase reactants though there is no documented evidence to substantiate this claim. Thus, the idea that these levels could not be accurately measured during an acute event has since been incorporated into medical dogma without being further validated [2-4,6-9]. Given the fact that these proteins are vitamin K dependant, late testing requires temporary interruption of oral anticoagulant therapy for at least 10 days and, in some cases, bridging anticoagulation with alternative agents such as low molecular weight heparin (LMWH) with the inherent costs and inconvenience.

Our group previously published data on 254 patients with acute VTE in whom proteins C and S were determined within 24 hours of diagnosis before the initiation of oral anticoagulation[10]. Abnormal results were repeated at least 3 months after starting treatment and at least 14 days after stopping anticoagulant therapy. This study identified that the initial false positive rate for all protein C and protein S tests was only 2.2% and almost 98% of patients had correct results as assessed at diagnosis. A criticism of this study was that we did not repeat the normal results to ensure that these were not false negatives. In the current study we sought to verify patients with initially normal protein C and S determinations were, in fact, true normals by repeating their testing after anticoagulant therapy was discontinued.

## Methods

### Patients

We studied consecutive patients referred to the outpatient thromboembolism clinics at a university hospital with a diagnosis of acute symptomatic VTE objectively confirmed by previously described criteria[11,12] All patients were at least 18 years old. Exclusion criteria were: previous venous thromboembolic events, therapy with low molecular weight heparin for >48 h prior to testing, abnormal baseline international normalized ratio or activated partial thromboplastin time (aPTT), active malignancy, VTE secondary to transient risk factor, pregnancy, presentation in the first six weeks postpartum, known chronic liver disease (defined as persistent elevation above normal levels of alanine aminotransferase or gamma glutamyl transferase) and oral anticoagulant therapy (warfarin) in the previous 2 weeks.

### Thrombophilia Testing

Samples for thrombophilia testing were drawn prior to the initiation of oral anticoagulant therapy and within 24 h of diagnosis of venous thromboembolism for all patients. Blood samples were collected in 0.109 M (3.2%) sodium citrate tubes (Becton_Dickinson, NJ, USA). Plasma was obtained and samples were spun at 2000 g for 20 minutes twice at 20°C and then frozen at -70°C and tested in batches on a weekly basis. Protein C was measured on an ACL 9000 Coagulometer (Instrumentation Laboratories, Lexington, MD, USA) using IL Test ProClot (Instrumentation Laboratory) reagent. Protein S was measured on an ACL 3000 Coagulometer by the method of IL-Test protein S (Instrumentation Laboratory). The IL Test ProClot kit is a functional clotting protein C assay, based on the prolongation of the aPTT in the presence of activated protein C. The IL Test protein S kit is a functional clot based assay of free protein S. If a patient's initial testing of protein C or protein S were normal, the results were repeated after at least 6 months of anticoagulation therapy provided the patient had discontinued warfarin for at least 14 days. Patients who had initially abnormal tests were excluded from this study. At the time of study our laboratory normal range for protein C was 0.72-1.23 U/ml and the normal range for protein S was 0.60-1.60 U/ml. The previously published study used the same methods for PC and PS determination as the current study.

### Statistical analysis

The proportion of patients who tested abnormal on the second test was determined and confidence intervals for proportions were determined by the Wilson's score method[13] using OpenEpi version 2.3[14]. For comparison purposes data from the previous study was included and analyzed in the same way. Comparison between groups was done using χ^2 ^tests with 2-sided p-values < 0.05 considered to be statistically significant. A post-hoc analysis to determine the appropriateness of our sample to detect the observed proportion of false positive results on repeat testing showed that a sample size of 100 would be adequate to detect a 3.7% upper confidence bound at the 97.5% level of significance assuming a true sample proportion of 0.

## Results

Ninety-nine patients with normal protein C and S determinations during the acute phase of VTE diagnosis were included in the study. There were 47 men and 52 women with a median age of 56 years (range 21-87). 50 patients had a diagnosis of deep vein thrombosis, 45 had a diagnosis of pulmonary embolism and 4 had concomitant deep vein thrombosis and pulmonary embolism. All patients had idiopathic VTE events. On repeat testing after anticoagulant therapy discontinuation, none of the 99 patients had an abnormal result of either protein C or S (0%, 95% CI 0-3.7%) (Table 1). Data from our previous study on patients with initially abnormal protein C and S determination was then used for comparison. Among patients who initially had abnormally low results, 40% (95%CI 35.4-84.8%) were confirmed to have low protein C and 63.6% (95%CI 16.8-68.7%) low protein S on repeated testing (Table 1). The difference of proportions between groups was statistically significant (χ^2 ^p-value < 0.001).

**Table 1:** Results of protein C and protein S testing performed at diagnosis and on repeat testing after discontinuing anticoagulation therapy in patients with venous thromboembolism.

	Protein C		Protein S	
	**Current study** **(N = 99)**	**Previous study**[16] **(N = 254)**	**Current study** **(N = 99)**	**Previous study**[16]**(N = 254)**

Initial normal result	99	242	99	242

Initial abnormal result	0	12	0	12

Repeat abnormal result	0	4^a^	0	6^a^

Proportion Abnormal on Repeat (95% CI)	0% (0-3.7%)^b^	40% (35.4-84.8%) ^b^	0% (0-3.7%)^b^	63.9% (16.8-68.7%) ^b^

CI confidence interval

^a ^Repeat testing was done only if initial test abnormal

^b ^P-value < 0.001 for comparison between current and previous study

## Discussion

Thrombophilia screening, including protein C and S determinations is an important part of the diagnostic investigation for idiopathic VTE in order to determine etiology, duration of anticoagulation, and to guide family testing [7,8,15]. It is widely believed that assays for natural coagulation inhibitors cannot be performed during the acute phase of VTE due to a consumptive decrease in protein C and S leading to false positive testing. This assumption is based on the study by D'Angelo et al that identified a mean decrease in protein S activity in patients with acute VTE before warfarin therapy[5]. This transient state of acquired protein S deficiency was thought to be a result of increase in C4bBP levels secondary to inflammation. The study only examined 8 patients and provided only a mean protein S level rather than showing the proportion of patients that had a level below the reference range. The authors of this study commented that these results would need to be repeated before concluding that acute VTE falsely lowers levels of protein S. These results have not been replicated in any further studies and, to our knowledge, the only other studies investigating this issue are our previous study in 2006 and this follow up study.

Our previous study in 254 patients with acute VTE showed that 91% of patients had normal protein C and S levels and among those with abnormal initial tests 4/12 and 6/12 were proven to have PC and PS deficiency at a later date. This figure resulted in a sensitivity of 100% and specificity of 97% and 98% respectively with the caveat that patients with an initial normal test were not retested at a later date therefore we could not be sure of possible missed false negatives. The current study shows that our previous assumptions are correct and PC and PS testing can be done at the time of an acute VTE event with a high degree of specificity and sensitivity. Assuming a pre-test probability of 2% the negative likelihood ratio for PC testing is 0 (95% CI, 0%-3%) and for PS testing is 0 (95% CI, 0%-2%).

There is no indication from this series of patients that acute VTE alters the levels of protein C or S enough to affect the diagnosis of coagulation factor deficiency. Whereas genetic tests such as Factor V Leiden and the G20210A prothrombin gene variant can be tested at any time, coagulometric assays (such as protein C and S) cannot be performed in patients anticoagulated with vitamin K antagonists. Therefore, patients with normal protein C or S levels at the time of diagnosis can be effectively ruled out as having deficiency of these proteins and can therefore avoid the expense and inconvenience of repeat testing during VKA therapy interruption.

In conclusion, our current study in combination with our previous results show that testing protein C and S levels during the acute diagnosis of VTE is a valid approach. We suggest that thrombophilia testing be performed at the time of diagnosis of VTE, before VKA therapy is initiated. This is the most convenient time for testing and allows early identification of patients who may require more prolonged therapy or further screening of family members. Only those who have an initially abnormal test should have confirmatory testing repeated after a course of VKA therapy and may require LMWH bridging. In this study we did not assess whether other coagulation based assays, such as antithrombin, can be accurately tested at the time of acute VTE diagnosis. This would be an interesting avenue for future research. In any case, since warfarin does not affect antithrombin levels, testing for antithrombin deficiency is possible at a later date without the need for interrupting anticoagulation.

## Competing interests

The authors declare that they have no competing interests.

## Authors' contributions

LM was responsible for data collection and analysis and helped draft the manuscript. ALL was responsible for the statistical analysis and helped edit and revise the manuscript. MK conceived of the study, participated in the design and coordination and helped draft the final manuscript. JK was involved in extracting data from charts. MR and BM were involved in handling the patient samples and organization of lab testing. All authors read and approved the final manuscript.

## References

[B1] Weinmann EE Salzman EW Deep-vein thrombosis N Engl J Med 1994 331 1630 1641 10.1056/NEJM199412153312407 7772110

[B2] Lane DA Mannucci PM Bauer KA Bertina RM Bochkov NP Boulyjenkov V Inherited thrombophilia: Part 2 Thromb Haemost 1996 76 824 834 8971998

[B3] Lane DA Mannucci PM Bauer KA Bertina RM Bochkov NP Boulyjenkov V Inherited thrombophilia: Part 1 Thromb Haemost 1996 76 651 662 8950768

[B4] Bauer KA The thrombophilias: well-defined risk factors with uncertain therapeutic implications Ann Intern Med 2001 135 367 373 11529700 10.7326/0003-4819-135-5-200109040-00013

[B5] D'Angelo A Vigano-D'Angelo S Esmon CT Comp PC Acquired deficiencies of protein S. Protein S activity during oral anticoagulation, in liver disease, and in disseminated intravascular coagulation J Clin Invest 1988 81 1445 1454 10.1172/JCI113475 3284913 PMC442576

[B6] Cumming AM Shiach CR The investigation and management of inherited thrombophilia Clin Lab Haematol 1999 21 77 92 10.1046/j.1365-2257.1999.00210.x 10342066

[B7] Kearon C Crowther M Hirsh J Management of patients with hereditary hypercoagulable disorders Annu Rev Med 2000 51 169 185 10.1146/annurev.med.51.1.169 10774459

[B8] Seligsohn U Lubetsky A Genetic susceptibility to venous thrombosis N Engl J Med 2001 344 1222 1231 10.1056/NEJM200104193441607 11309638

[B9] Thomas RH Hypercoagulability syndromes Arch Intern Med 2001 161 2433 2439 10.1001/archinte.161.20.2433 11700155

[B10] Kovacs MJ Kovacs J Anderson J Rodger MA Mackinnon K Wells PS Protein C and protein S levels can be accurately determined within 24 hours of diagnosis of acute venous thromboembolism Clin Lab Haematol 2006 28 9 13 10.1111/j.1365-2257.2006.00746.x 16430453

[B11] Wells PS Anderson DR Rodger M Stiell I Dreyer JF Barnes D Excluding pulmonary embolism at the bedside without diagnostic imaging: management of patients with suspected pulmonary embolism presenting to the emergency department by using a simple clinical model and d-dimer Ann Intern Med 2001 135 98 107 11453709 10.7326/0003-4819-135-2-200107170-00010

[B12] Wells PS Anderson DR Rodger M Forgie M Kearon C Dreyer J Evaluation of D-dimer in the diagnosis of suspected deep-vein thrombosis N Engl J Med 2003 349 1227 1235 10.1056/NEJMoa023153 14507948

[B13] Newcombe RG Two-sided confidence intervals for the single proportion: comparison of seven methods Stat Med 1998 17 857 872 10.1002/(SICI)1097-0258(19980430)17:8<857::AID-SIM777>3.0.CO;2-E 9595616

[B14] Dean AG Sullivan KM Soe MM OpenEpi: Open Source Epidemiologic Statistics for Public Health. [2.3] 2009 Atlanta, Rollins School of Public Health, Emory University Ref Type: Computer Program

[B15] Crowther MA Kelton JG Congenital thrombophilic states associated with venous thrombosis: a qualitative overview and proposed classification system Ann Intern Med 2003 138 128 134 12529095 10.7326/0003-4819-138-2-200301210-00014

[B16] Kovacs MJ Kovacs J Anderson J Rodger MA Mackinnon K Wells PS Protein C and protein S levels can be accurately determined within 24 hours of diagnosis of acute venous thromboembolism Clinical & Laboratory Haematology 2006 28 9 13 10.1111/j.1365-2257.2006.00746.x 16430453

